# Supplementary material for: Testing the limits of pheromone stigmergy in high-density robot swarms
Source: R Soc Open Sci. 2019 Nov 6;6(11):190225. doi: 10.1098/rsos.190225 (PMC6894587; doi:10.1098/rsos.190225)
Supplement: Supplementary figures - different pheromone decay constants; different sized arena; different pheromone deposition behaviour [file rsos190225supp1.pdf]

Testing the limits of pheromone stigmergy in spatially constrained robotic swarms

Supplementary material

Figure S1. Performance of **simulated** robotic swarms, pheromone decay constant $\boldsymbol{r} = \mathbf{0.005}$, in terms of (1) area coverage and (2) proportion of arena visited at least once, for N=1, 4, 16, 64, 144, 256, 324, 400. See section 3.4 of the methods for a description of these metrics. Median and lower and upper quartiles are shown for 30 simulations per group size.

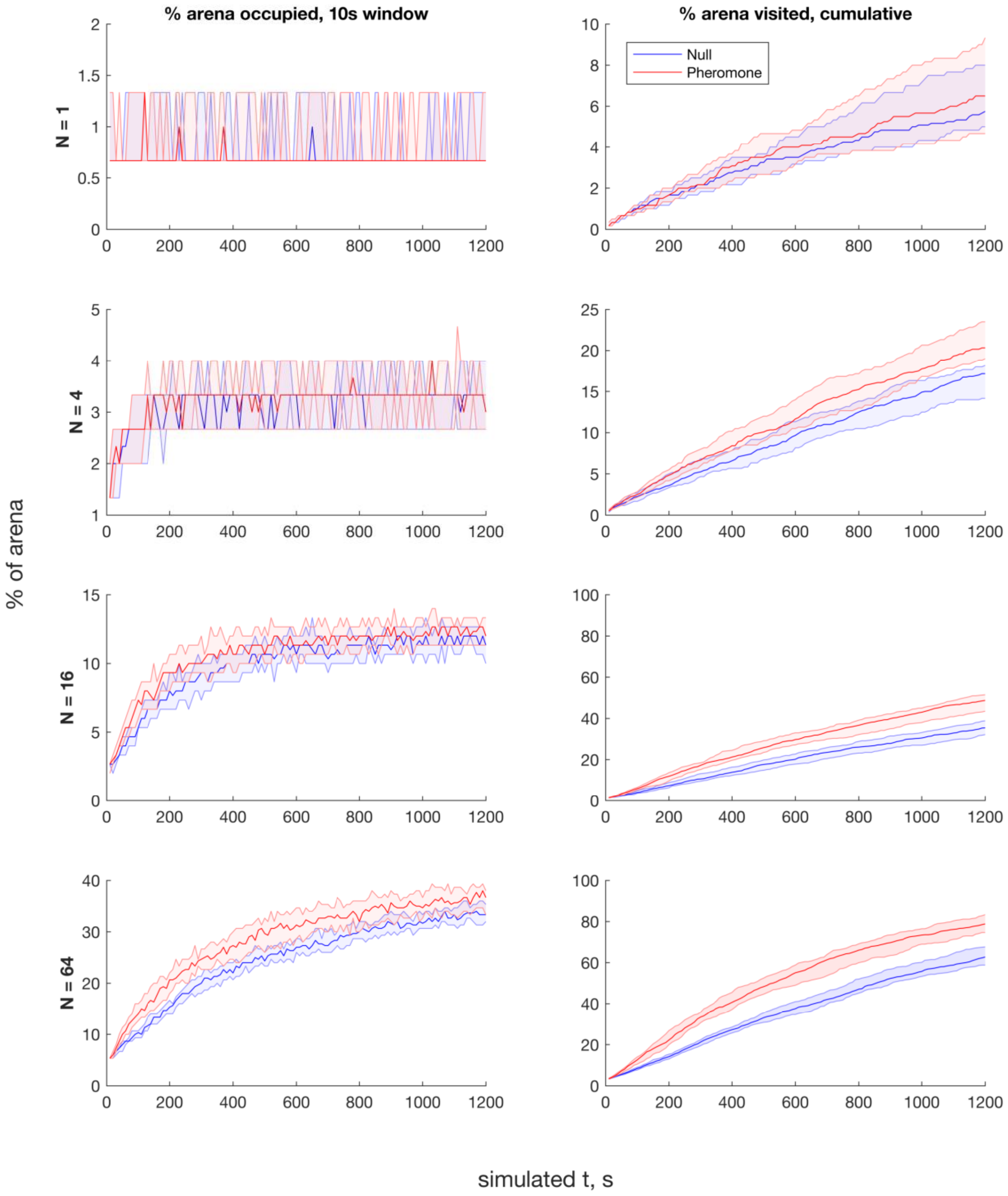

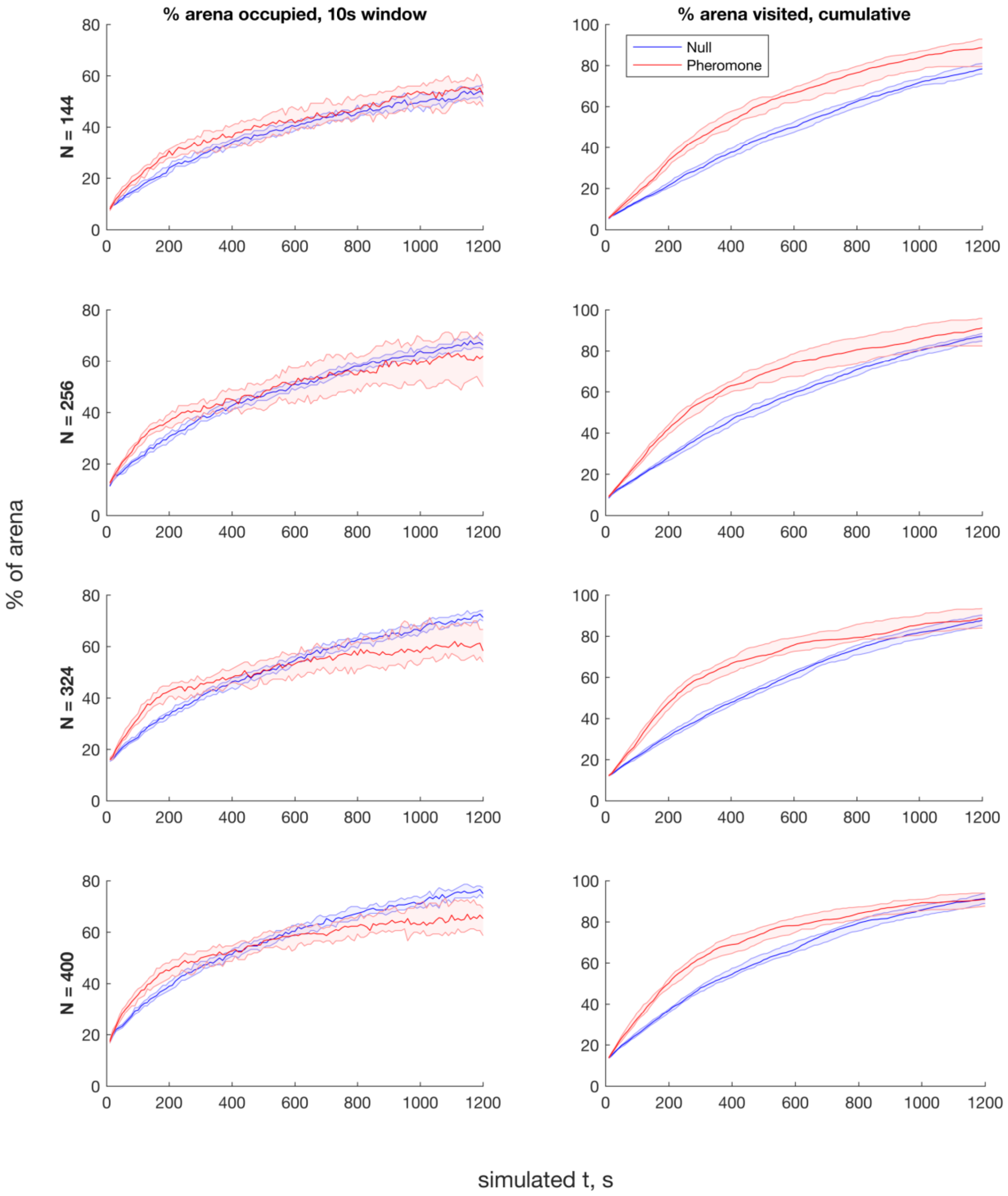

% arena occupied, 10s window
% arena visited, cumulative
Null
Pheromone
N = 144
N = 256
N = 324
N = 400
% of arena
simulated t, s

Figure S2. Performance of **simulated** robotic swarms, pheromone decay constant $\boldsymbol{r} = \mathbf{0.010}$ (i.e. twice as fast decay as Figure S1).

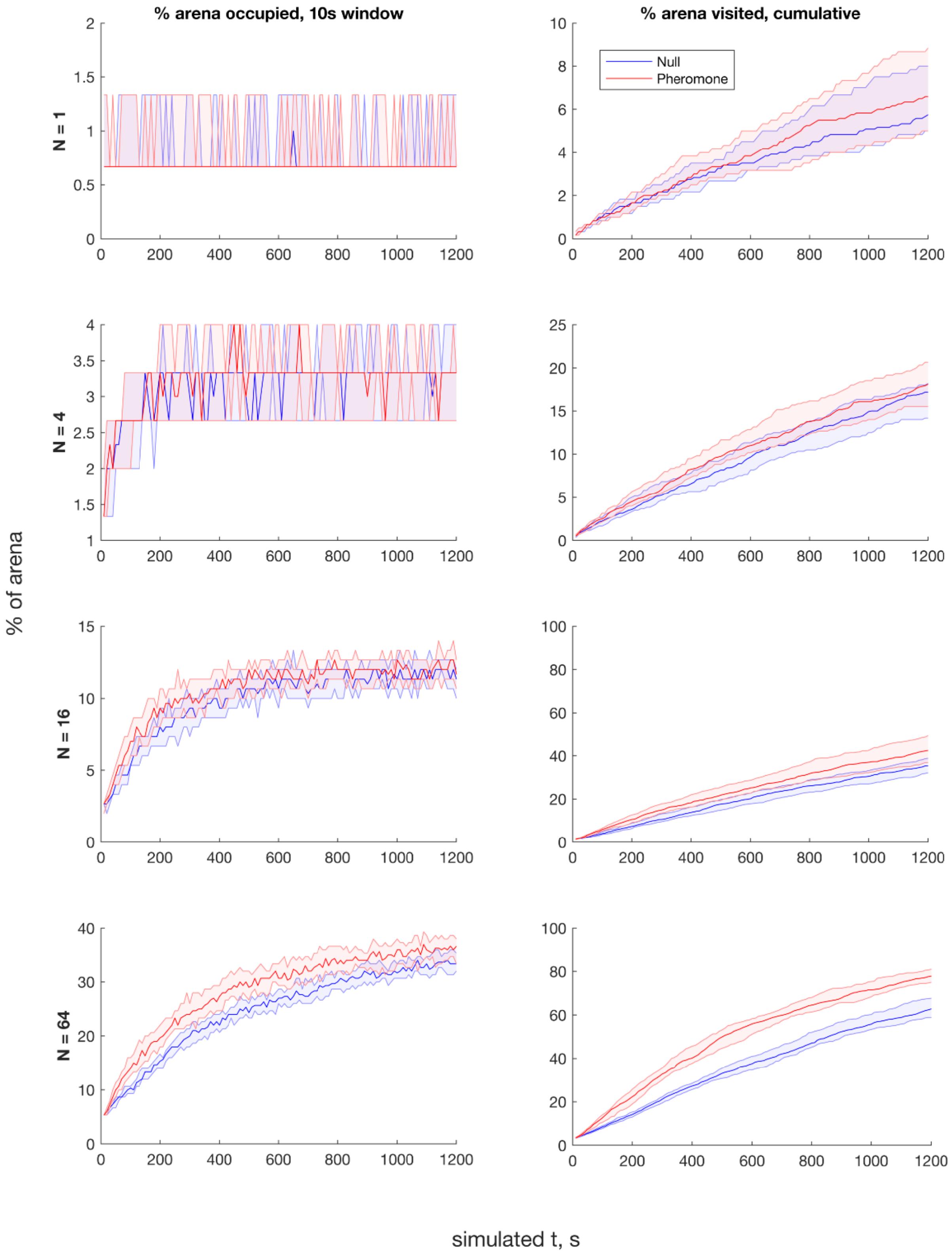

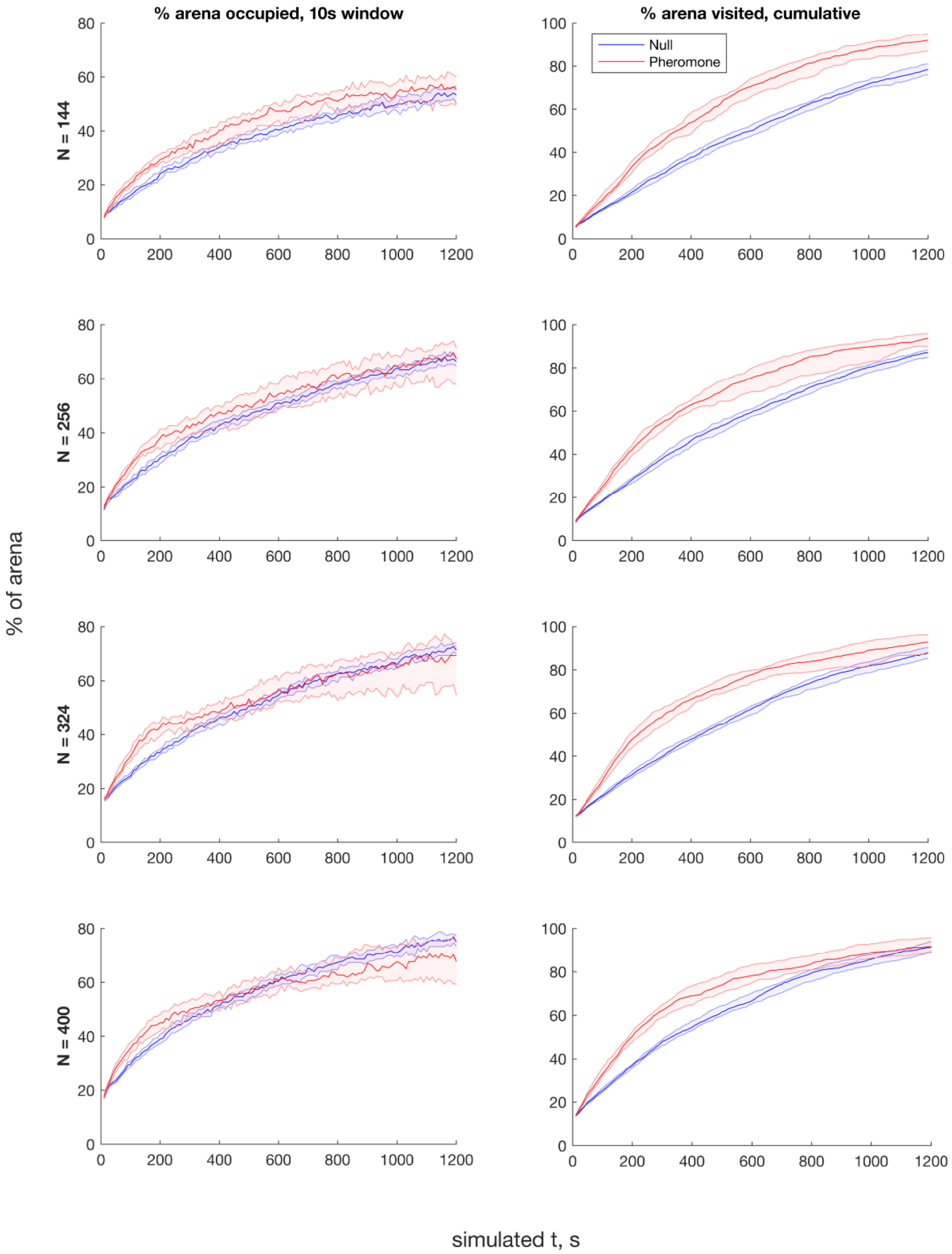
% arena occupied, 10s window
% arena visited, cumulative
Null
Pheromone
N = 144
N = 256
N = 324
N = 400
% of arena
simulated t, s

Figure S3. Performance of **simulated** robotic swarms, pheromone decay constant $\boldsymbol{r} = \mathbf{0.0025}$ (i.e. half as fast decay as Figure S1).

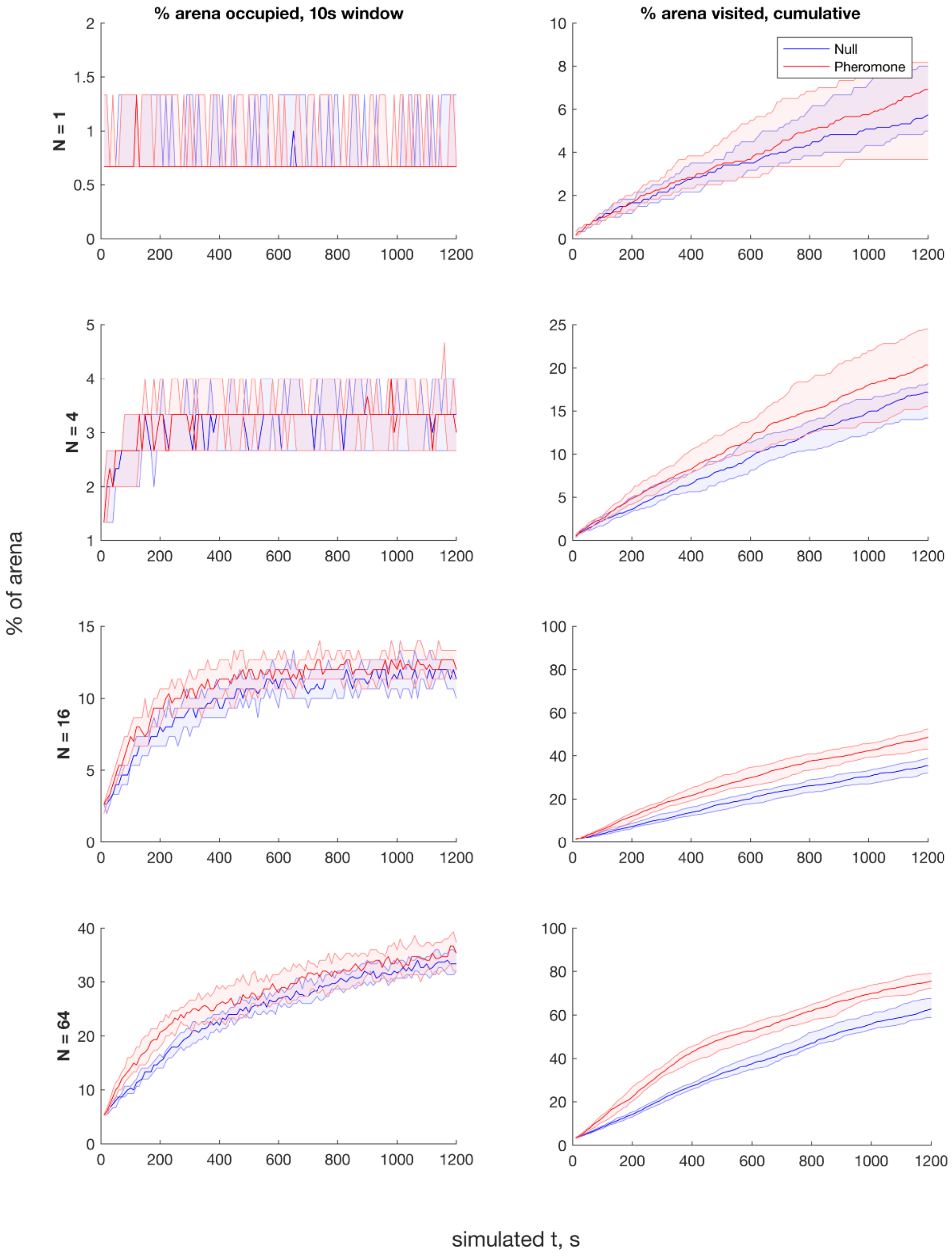

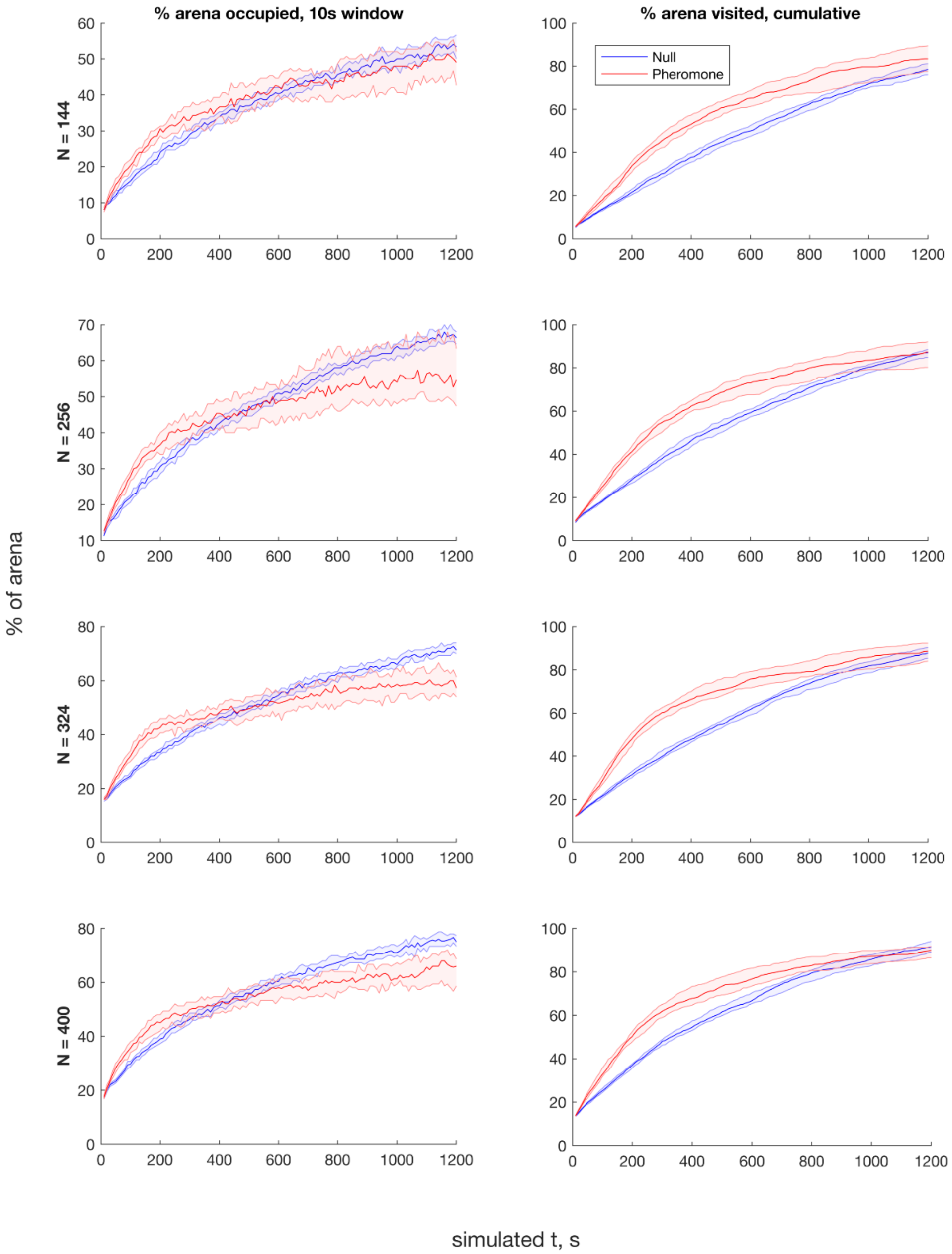

% arena occupied, 10s window
% arena visited, cumulative
Null
Pheromone
N = 144
N = 256
N = 324
N = 400
% of arena
simulated t, s

Figure S4. Performance of **simulated** robotic swarms, arena size $3m^2$ (i.e. half the area of $6m^2$ arena in Figures S1-3), but same density (half the number of Kilobots). Pheromone decay constant $\boldsymbol{r} = \mathbf{0.005}$.

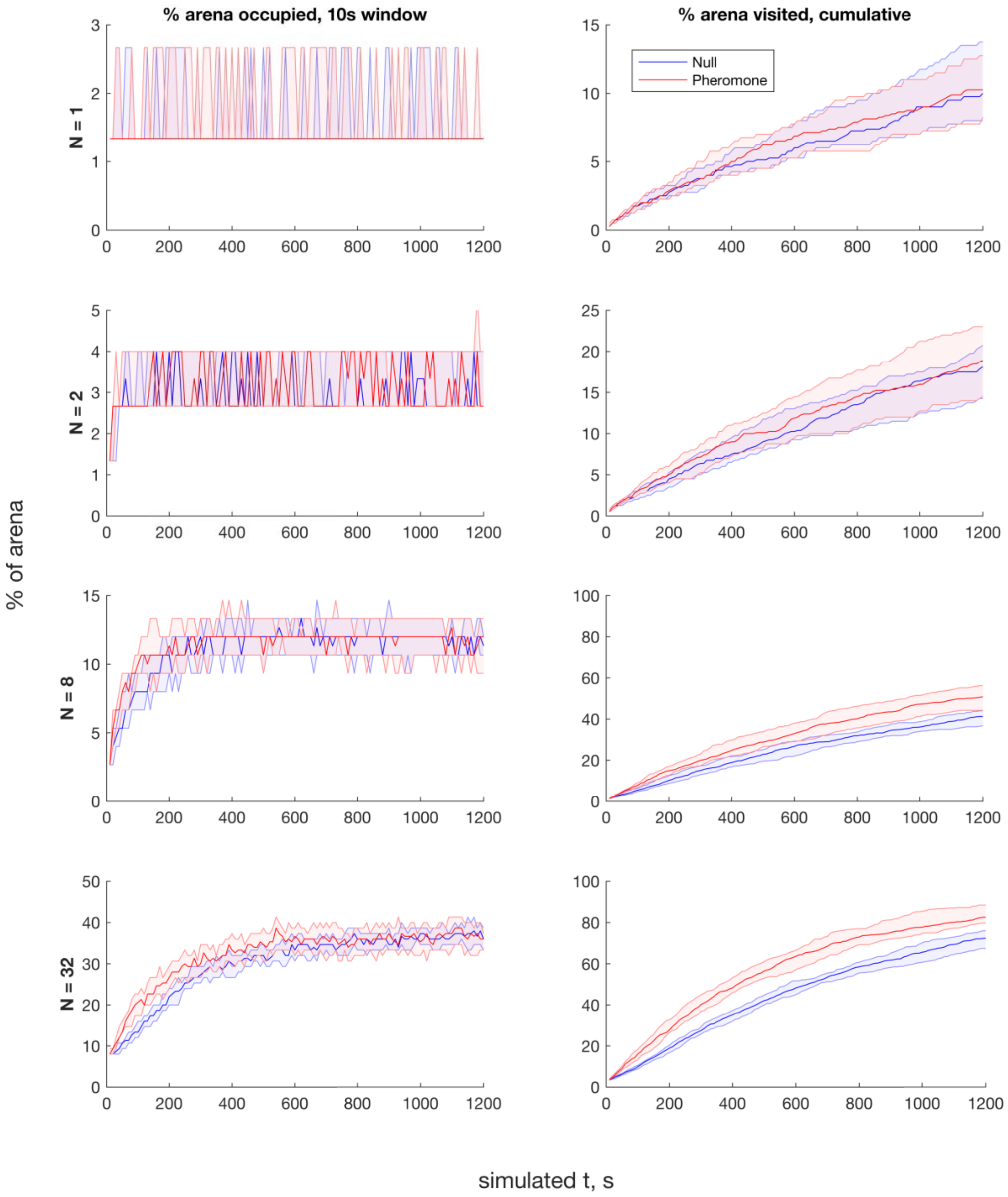

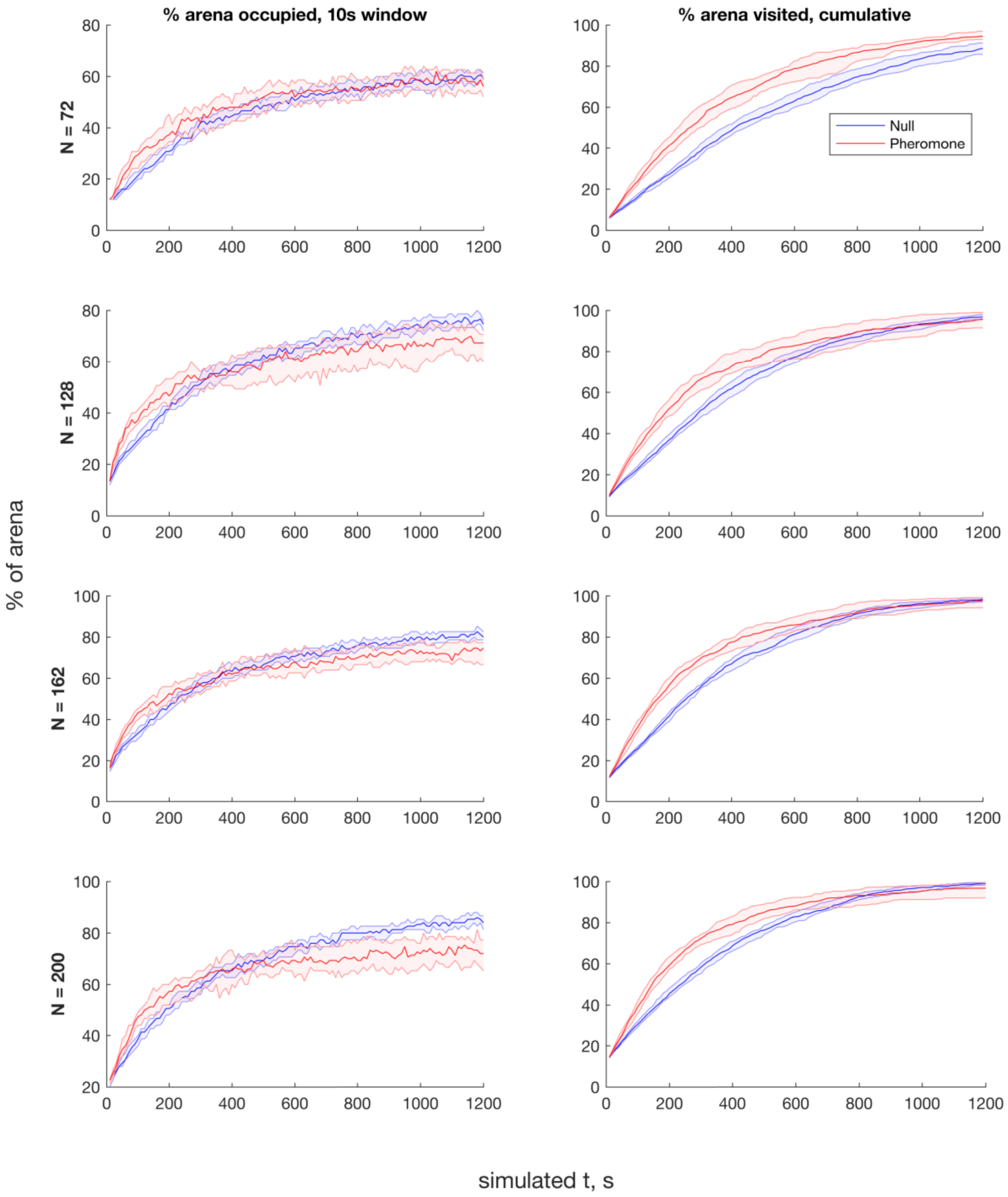

% arena occupied, 10s window
% arena visited, cumulative
N = 72
N = 128
N = 162
N = 200
Null
Pheromone
% of arena
simulated t, s

Figure S5. Performance of **simulated** robotic swarms, arena size 3m^2 (i.e. half the area of 6m^2 arena in Figures S1-3), but same density (half the number of Kilobots). Pheromone decay constant $\boldsymbol{r} = \mathbf{0.010}$.

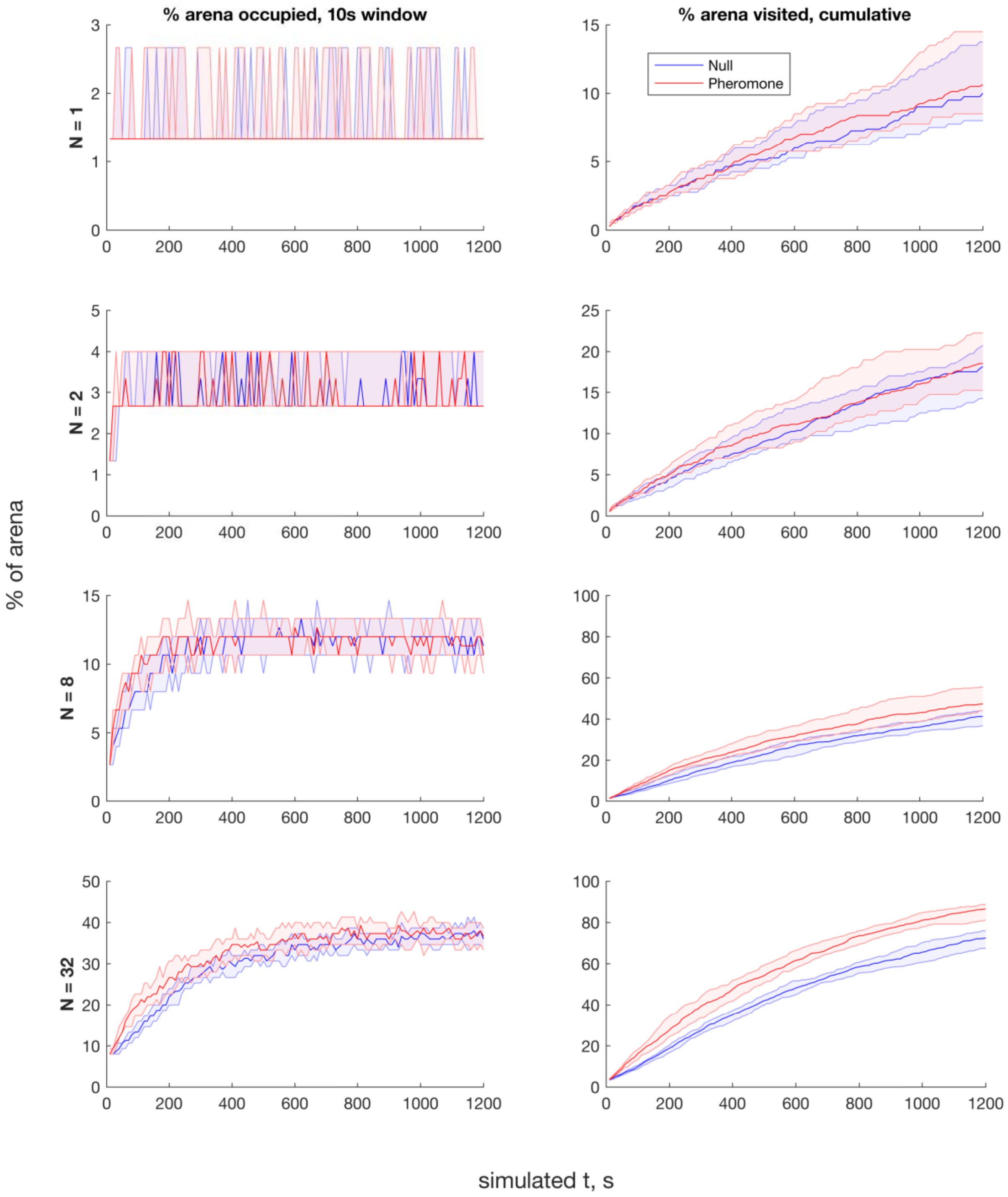

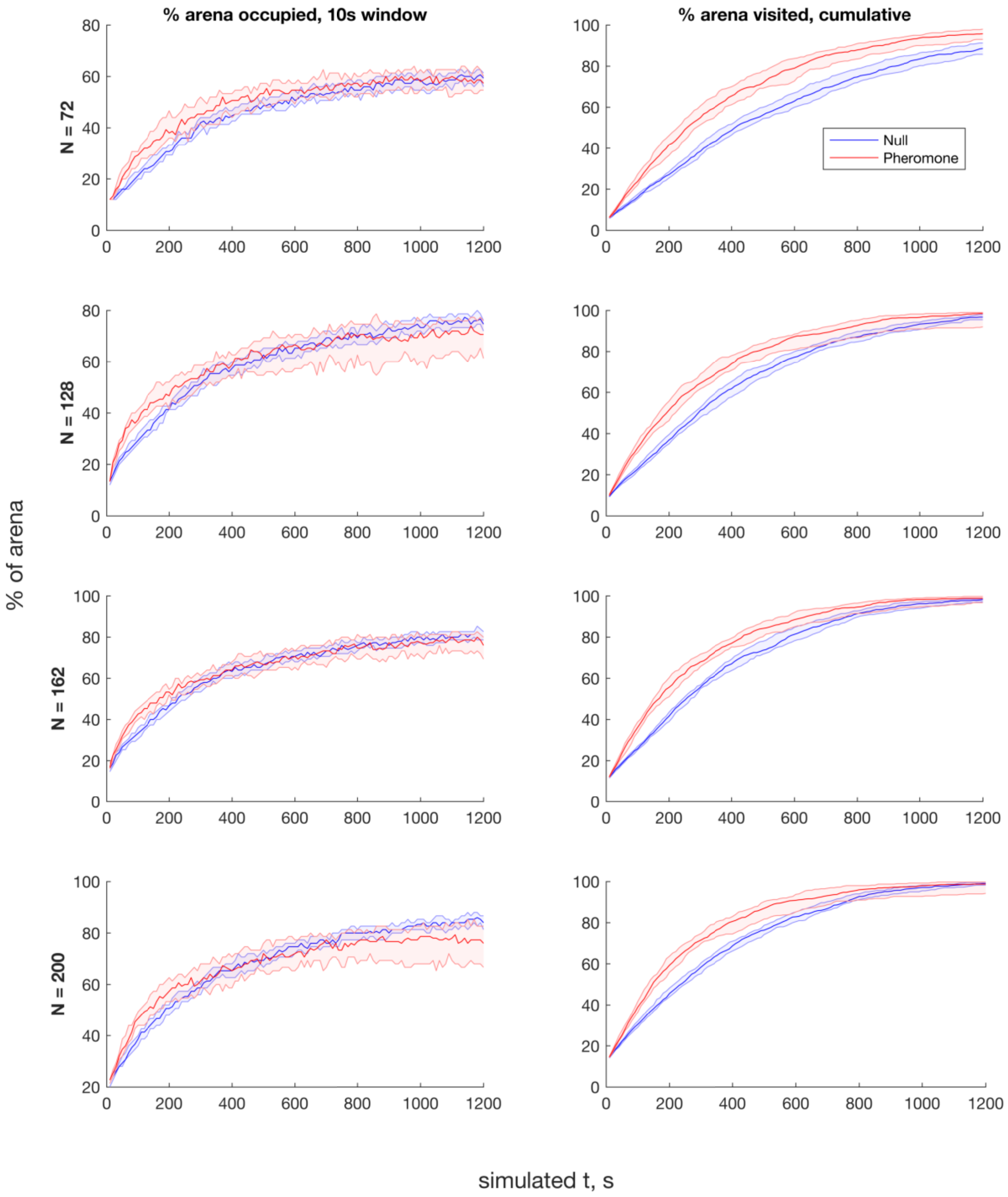

% arena occupied, 10s window
% arena visited, cumulative
N = 72
N = 128
N = 162
N = 200
% of arena
Null
Pheromone
simulated t, s
0
200
400
600
800
1000
1200
20
40
60
80
100

Figure S6. Performance of **simulated** robotic swarms, arena size 3m^2 (i.e. half the area of 6m^2 arena in Figures S1-3), but same density (half the number of Kilobots). Pheromone decay constant $\boldsymbol{r} = \mathbf{0.0025}$.

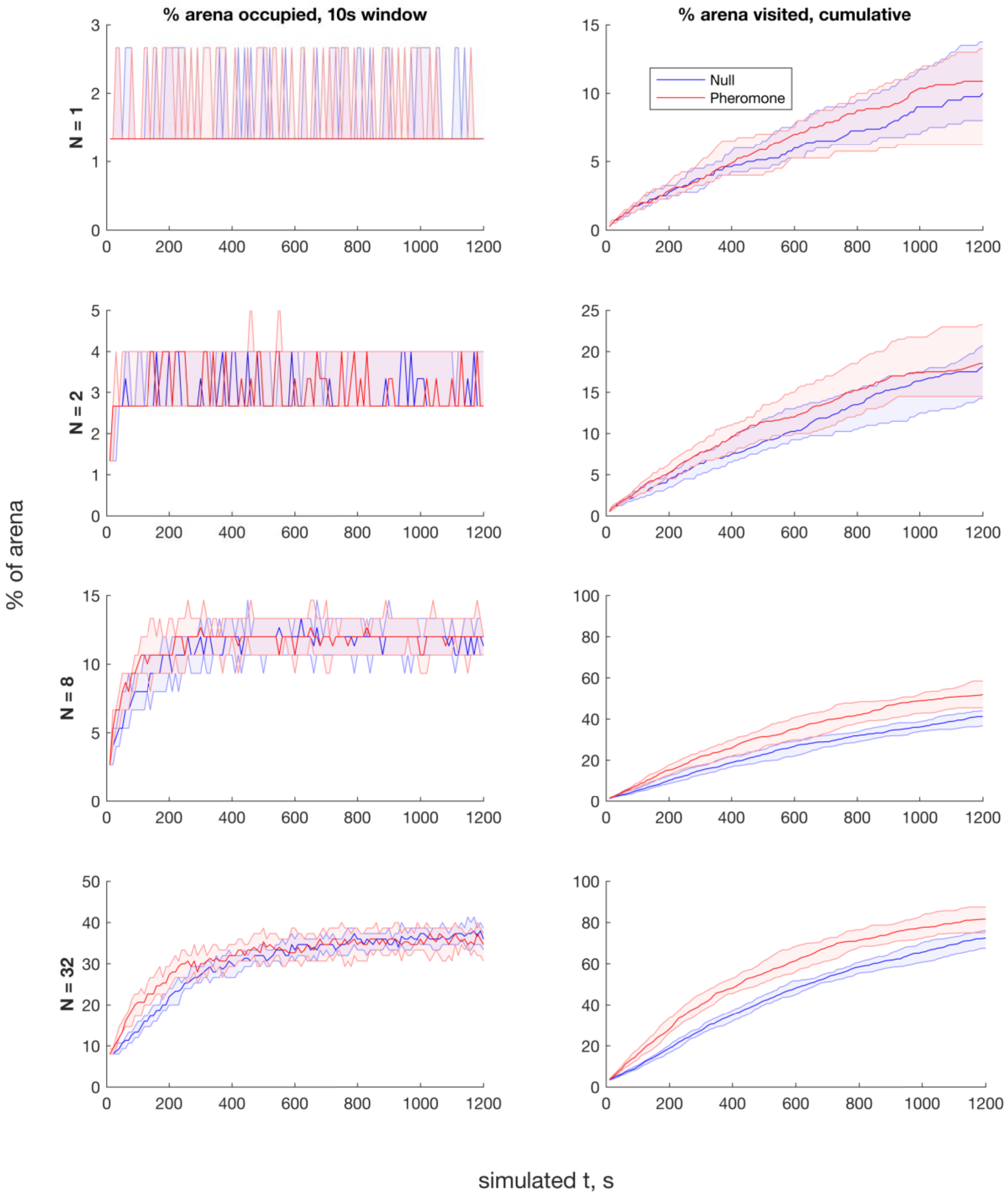

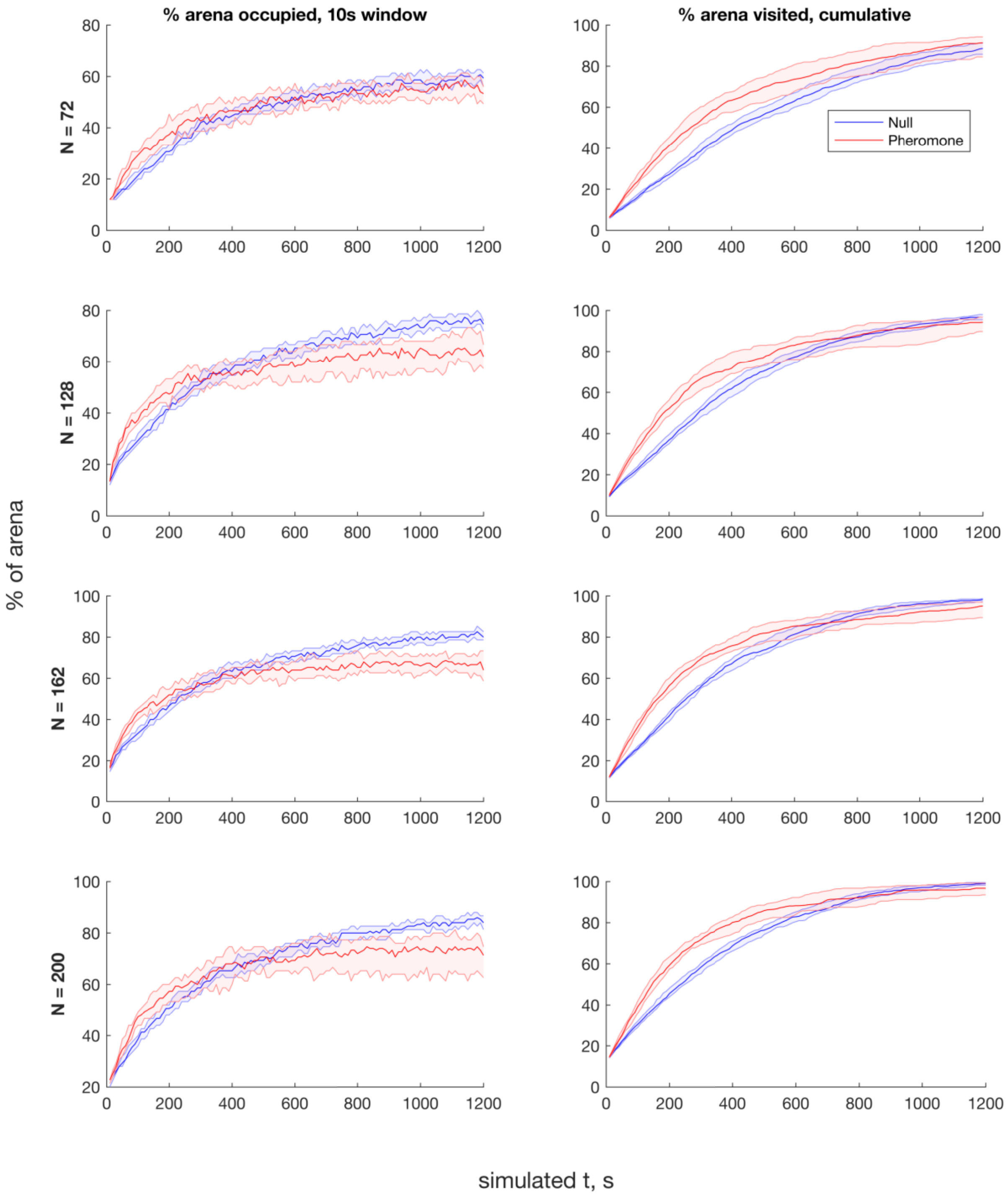

% arena occupied, 10s window
% arena visited, cumulative
N = 72
N = 128
N = 162
N = 200
Null
Pheromone
% of arena
simulated t, s

Figure S7. Performance of **simulated** robotic swarms, arena size 6m^2, pheromone only laid **below threshold of 0.75** (1 full saturation). Pheromone decay constant $\boldsymbol{r} = \mathbf{0.005}$.

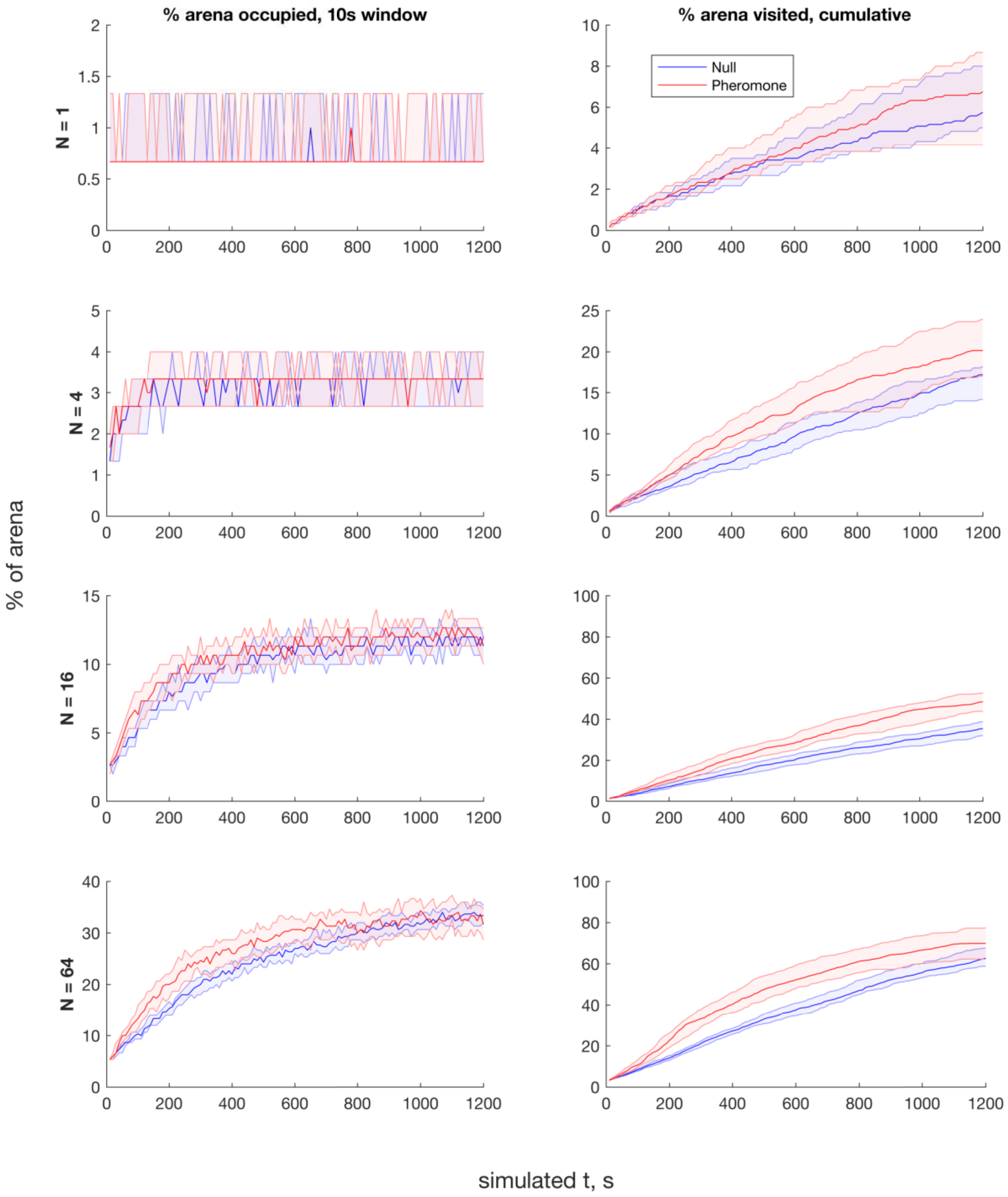

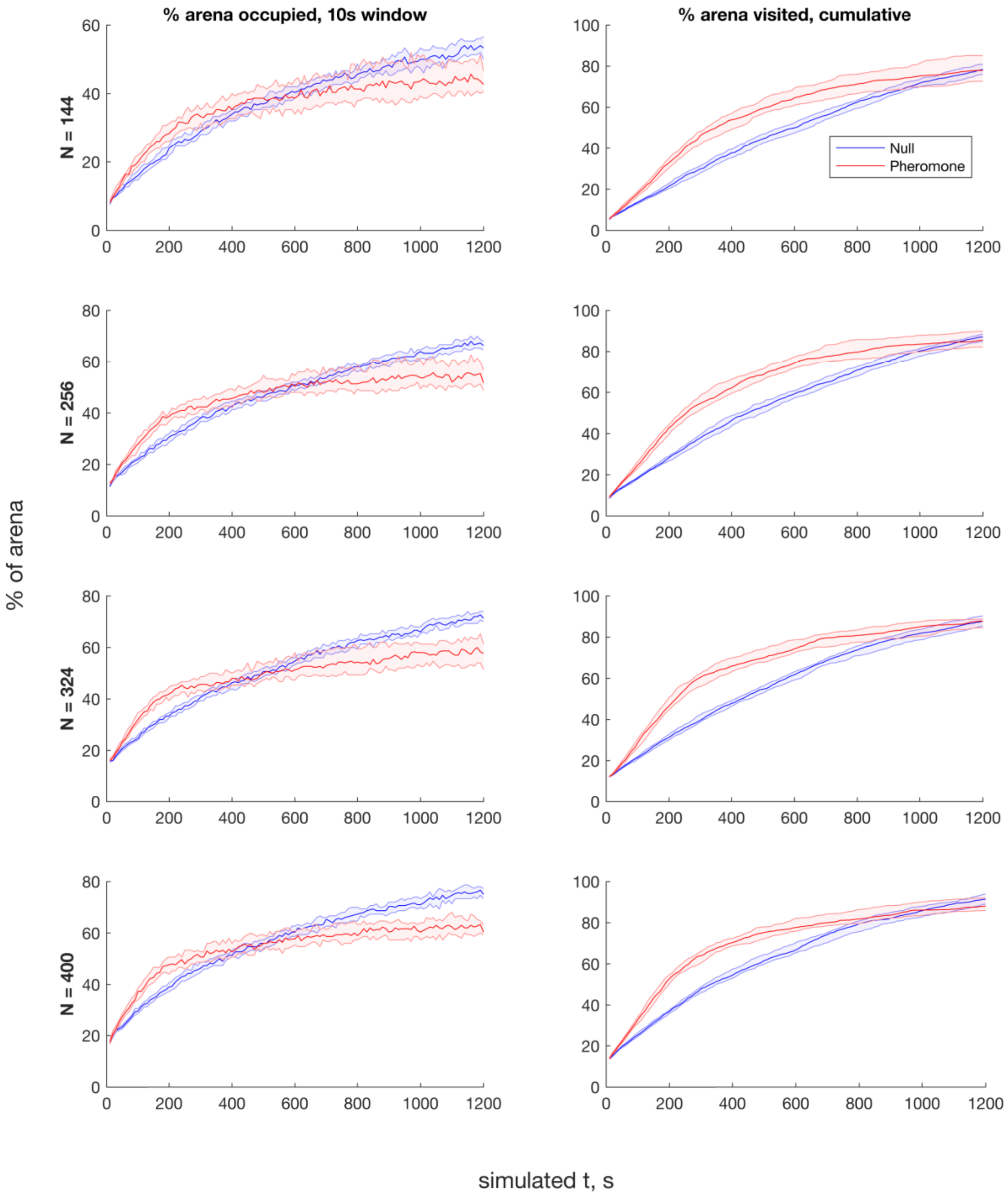
% arena occupied, 10s window
% arena visited, cumulative
N = 144
N = 256
N = 324
N = 400
Null
Pheromone
% of arena
simulated t, s

Figure S8. Performance of **simulated** robotic swarms, arena size 6m^2, pheromone only laid **below threshold of 0.75** (1 full saturation). Pheromone decay constant $\boldsymbol{r} = \mathbf{0.010}$.

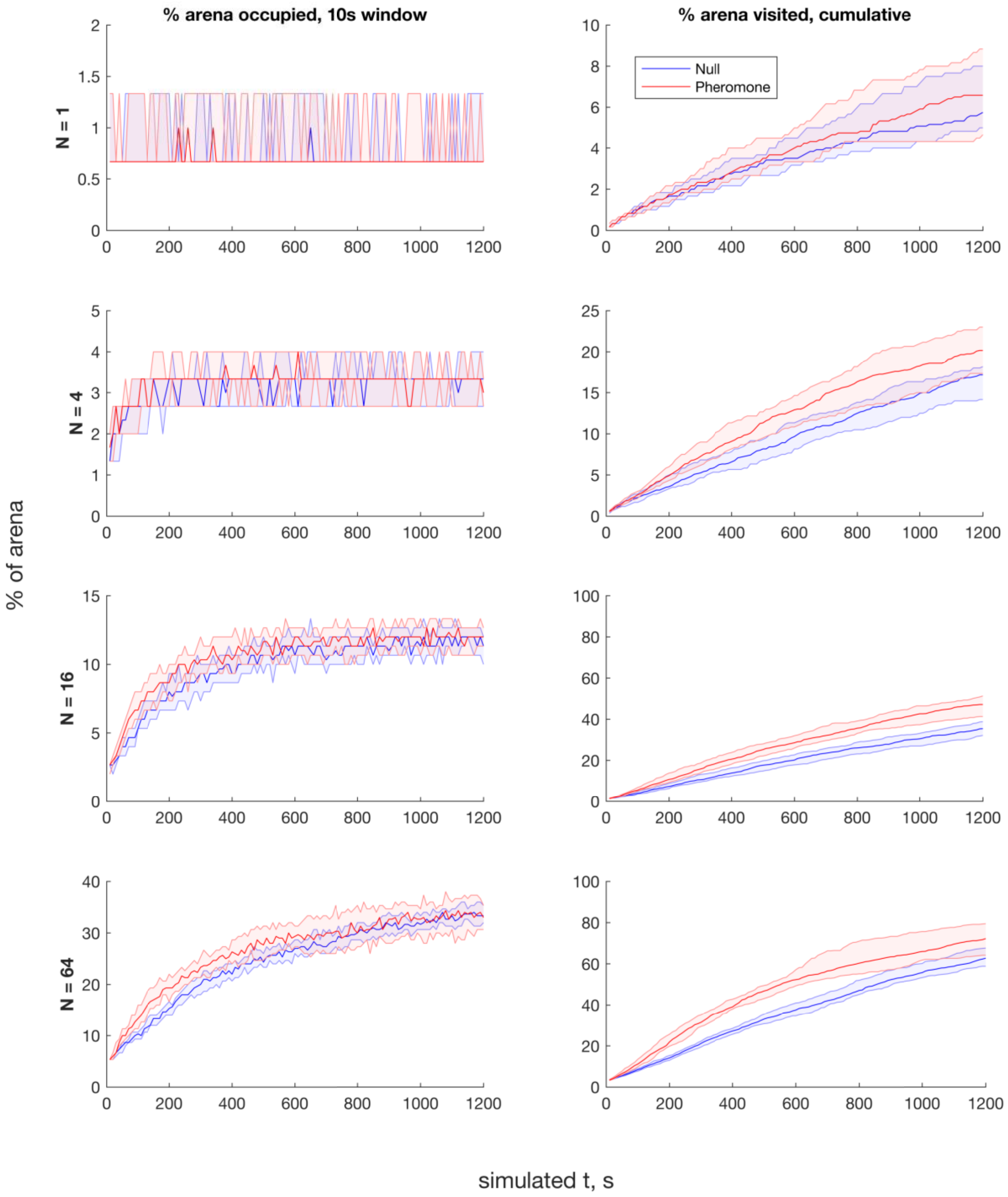

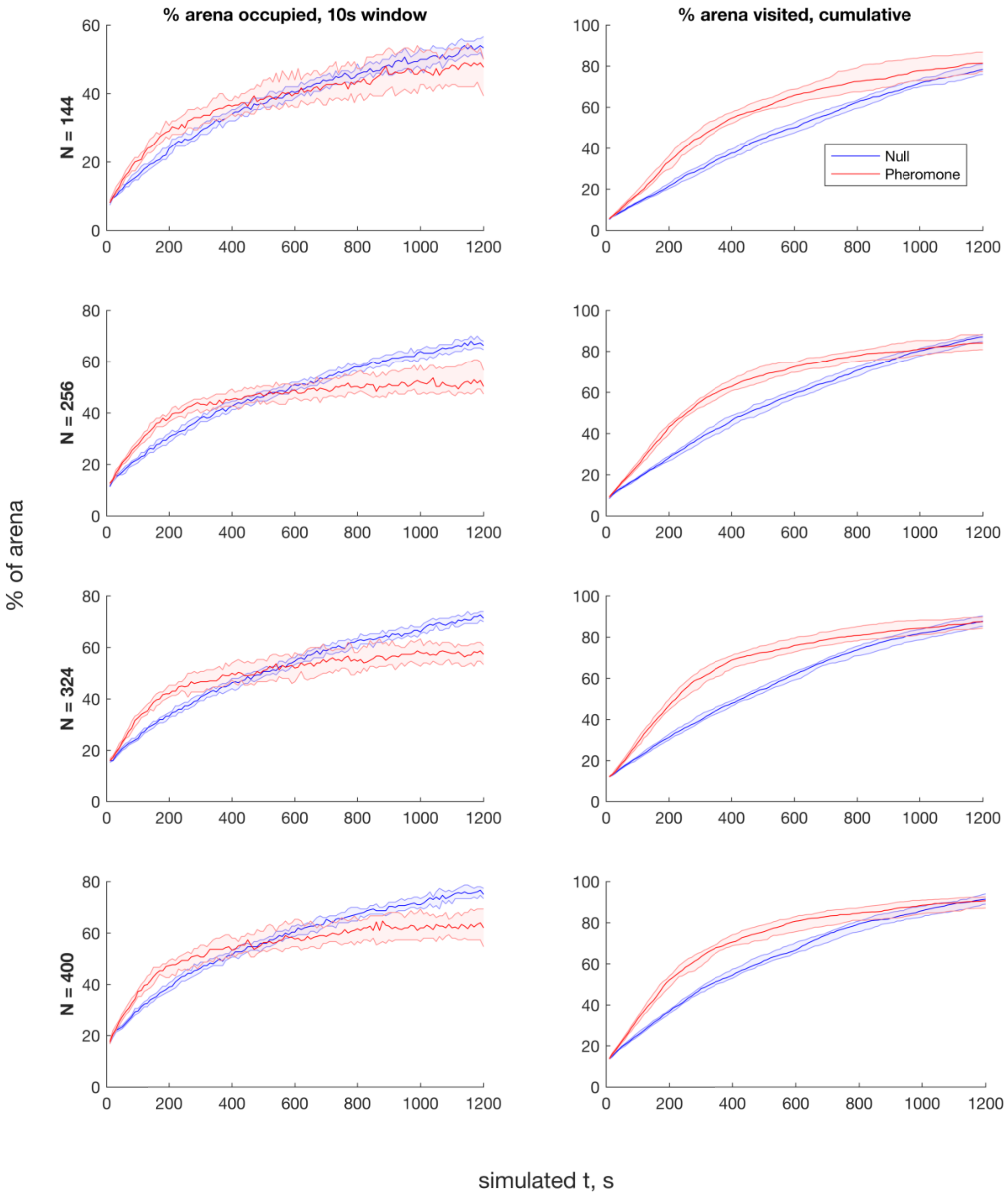

% arena occupied, 10s window
% arena visited, cumulative
N = 144
N = 256
N = 324
N = 400
Null
Pheromone
% of arena
simulated t, s

Figure S9. Performance of **simulated** robotic swarms, arena size 6m^2, pheromone only laid **below threshold of 0.75** (1 full saturation). Pheromone decay constant $r = 0.0025$.

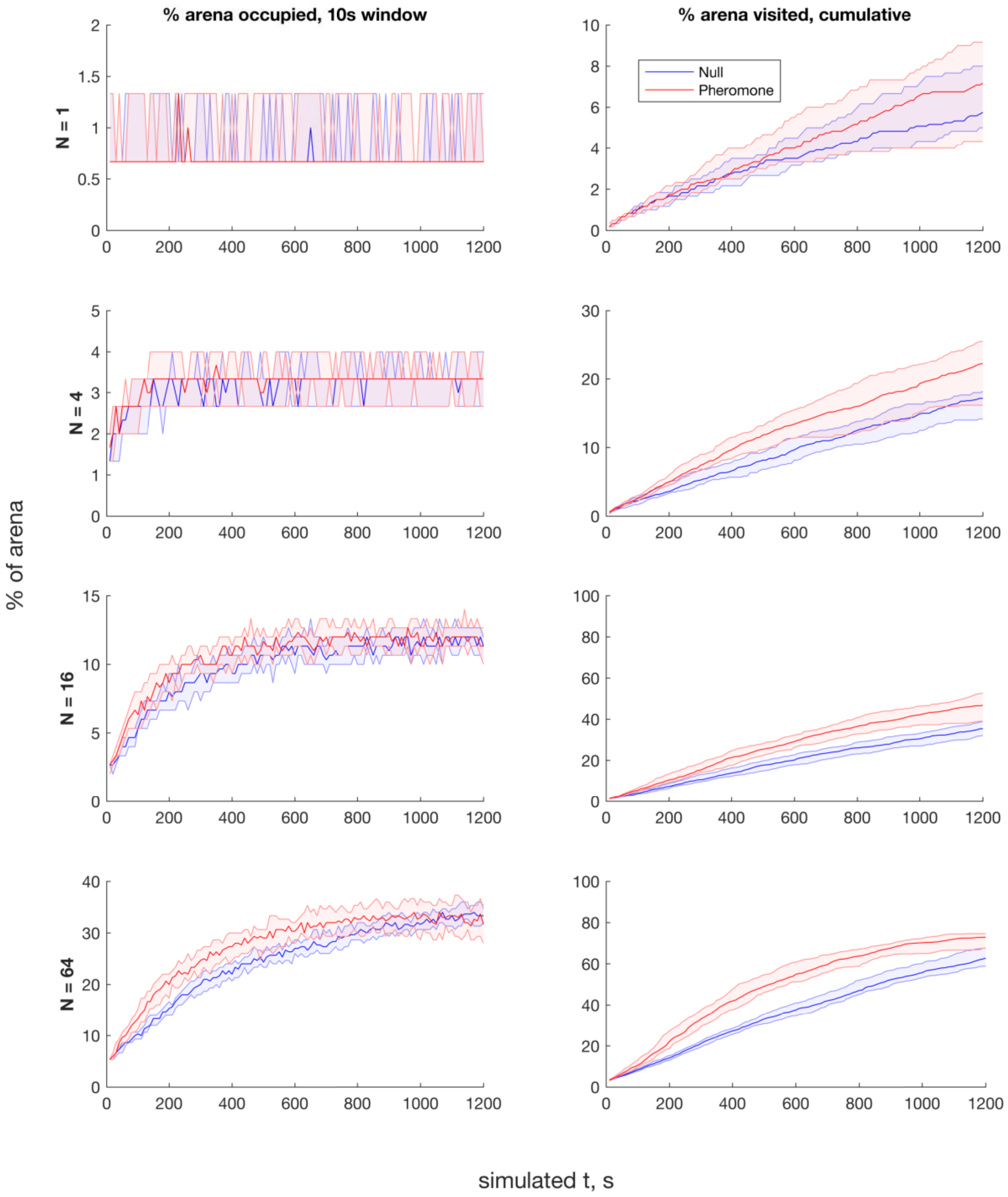

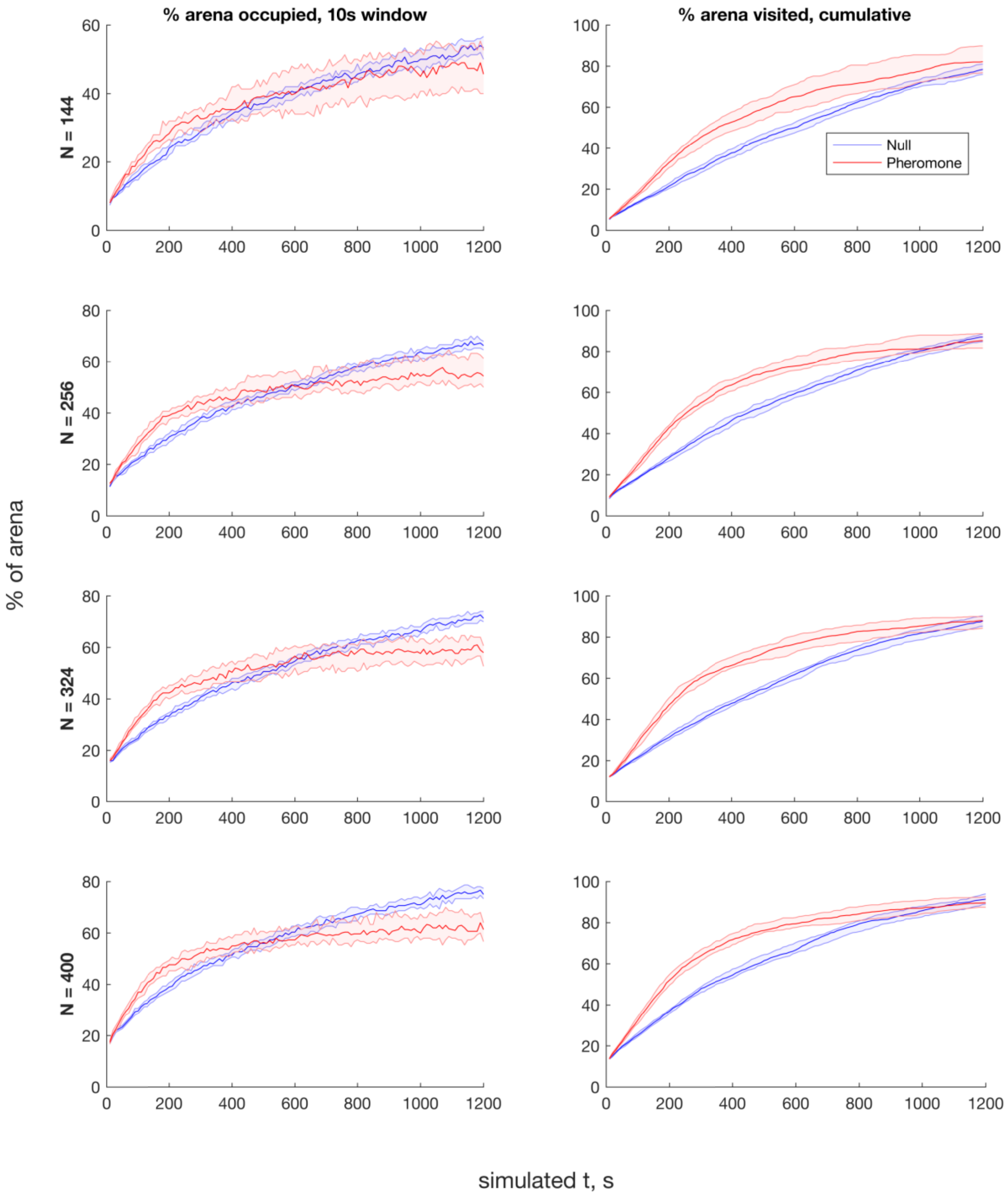

% arena occupied, 10s window
% arena visited, cumulative
N = 144
N = 256
N = 324
N = 400
Null
Pheromone
% of arena
simulated t, s
