# Supplementary material for: Testing the limits of pheromone stigmergy in high-density robot swarms
Source: R Soc Open Sci. 2019 Nov 6;6(11):190225. doi: 10.1098/rsos.190225 (PMC6894587; doi:10.1098/rsos.190225)
Supplement: Supplementary code - Kilobot controller [file rsos190225supp3.pdf]

ESM 3 – Robot controller code

Simulator code

The below code is written for the ‘Kilobox’ simulator, using the ‘stigmergy’ branch, available here:

https://bitbucket.org/siteks/kilobox

```
void Stigmergy_example::setup()
{
    last_update     = kilo_ticks;
    avoid_timer     = kilo_ticks;
    enable_pheromone();
}
void Stigmergy_example::loop()
{
    if (kilo_ticks > last_update + 16) //one 'tick' = 30ms, or 32 (33) ticks per second
    {
        last_update = kilo_ticks;

        //------------------------Timers for random walk and avoidance behaviors------
        if (random_walk==1){
            if (kilo_ticks > random_timer + random_duration){
                behavior_complete=1;
            }
        }
        if (avoid_behavior==1){
            if (kilo_ticks > avoid_timer + random_duration){
                behavior_complete=1;
            }
        }

        if (behavior_complete==1){      //only sense when past move completed
        int16_t e = get_environment();

            //-----------------------------------Not in pheromone ------------
            if (e==0){
                random_walk=1;
                avoid_behavior=0;

                random_timer=kilo_ticks;
                behavior_complete=0;

                int random_number = rand_hard();
                int rounded = (random_number % 3);
                random_duration = (16*(rounded+1)); //1-3 secs decided here - 'motor planning'
            }
            //-----------------------------------Detect pheromone --------
            if (e==1){
                avoid_behavior=1;
                random_walk=0;

                avoid_timer=kilo_ticks;
                behavior_complete=0;

                int random_number = rand_hard();
                int rounded = (random_number % 3);
                random_duration = (16*(rounded+1))/2; //0.5-1.5 secs

            }
        }

        //---------- Avoidance and random walk behaviors ----------------------------------

        //---------- Random walk behavior ------------------------
        if (random_walk==1){

            set_color(RGB(0,1,0));

                // Generate an 8-bit random number (between 0 and 2^8 - 1 = 255).
                int random_number = rand_hard();
                // Compute the remainder of random_number when divided by 3.
                // This gives a new random number in the set {0, 1, 2}.
                int random_direction = (random_number % 3);

                if (random_direction != direction_before)
                {
                    direction_before=random_direction;

                    if (random_direction == 0)
                    {
                        spinup_motors();
                        set_motors(kilo_turn_left, 0);
                    }
                    else if (random_direction == 1)
                    {
                        spinup_motors();
                        set_motors(0, kilo_turn_right);
                    }
                    else if (random_direction == 2)
                    {
                        spinup_motors();
                        set_motors(kilo_straight_left, kilo_straight_right);
                    }

                }
```

```
        }

        if (avoid_behavior==1){

            set_color(RGB(0,0,1));

            if (kilo_ticks == avoid_timer){ //turn for one tick, i.e. 0.5s, at beginning of avoidance

                //turn_timer=1;
                int random_number = rand_hard();
                // Compute the remainder of random_number when divided by 2.
                // This gives a new random number in the set {0, 1}.
                int random_direction = (random_number % 2);

                if (random_direction==0){
                    spinup_motors();
                    set_motors(kilo_turn_left, 0); //randomly go left or right
                }else{
                    spinup_motors();
                    set_motors(0,kilo_turn_right);
                }
            }
        //----------------------------------Otherwise go straight ------------------------------
            spinup_motors();
            set_motors(kilo_straight_left, kilo_straight_right);
        }
    } //end Kilobot loop
```

Real robot code

For real Kilobots, the above code can be used where noted, with the addition of the following function for get_environment()

```
#include <kilolib.h>
#include <avr/interrupt.h>
#include <util/delay.h>
#include "macros.h"
#include <stdlib.h>
#include <string.h>

int get_environment() {
    int samples[16];
    int binary[16] = {0,0,0,0,0,0,0,0,0,0,0,0,0,0,0,0};
    int bin[16] = {0,0,0,0,0,0,0,0,0,0,0,0,0,0,0,0};
    cli();
    // ADC input 7 is ambient light sensor, conversion with prescale
    // of 8 = 1MHz. First conversion takes 25us, subsequent take 13us
    //
    // Take 16 samples at approx 500us intervals, this should encompass
    // one cycle of the DLP colour wheel.
    //
    //
    // Examinging the waveforms under different colours gives the following
    // patterns
    //
    // Y XXXxxxX_________
    // M XX____xx________
    // C Xxxxxxx_________
    // R xx______________
    // G XX______________
    // B XX______________
    //
    // With simple processing, it should be possible to reliably distinguish
    // M, Y/C, R/G/B
    // giving a four character alphabet (with black)
    adc_setup_conversion(7);
    for(int *p = samples; p < samples + 16; p++)
    {
        adc_start_conversion();
        _delay_us(500);
        *p = ADCW;
    }
    adc_trigger_high_gain();
    sei();
    int min = 1024;
    int max = 0;
    for(int i = 0; i < 16; i++)
    {
        if (samples[i] < min) min = samples[i];
        if (samples[i] > max) max = samples[i];
    }
    if ((max - min) < 10)
    {
        // Too narrow a range, assume to be no colour
        return 0;
    }
    // There are four apparent brightness levels, but they are not evenly spread
    // By examining the data, this gives a good basic threshold
    int thresh = min + (max - min) / 4;
    int count = 0;
    // Binarise
    for(int i = 0; i < 16; i++)
    {
        binary[i] = samples[i] >= thresh;
        count += binary[i];
    }
```

```
    // Find longest string of zeros. do this by looking for a 1->0 transition and
    // then counting from there, regarding buffer as circular
    int max_length = 0;
    int max_end = 0;
    for(int i = 0; i < 16; i++)
    {
        if ((binary[i] == 1) && (binary[(i+1)%16] == 0))
        {
            // Found start
            int length = 0;
            for(int j = 0; j < 16; j++)
            {
                if (binary[(i+j+1) % 16] == 0)
                    length++;
                else if (length > max_length)
                {
                    max_length = length;
                    max_end = (i+j) % 16;
                    length = 0;
                }
            }
            break;
        }
    }

    for(int i = 0; i < 16; i++)
    {
        bin[i] = binary[(max_end + i + 1) % 16];
    }

    if (count == 0)
        // No colour
        return 0;
    if (count < 4)
        // Primary colour, R, G, or B
        return 1;
    if ((count >= 4) && (bin[4] == 0))
        // Double pulse - Magenta
        return 2;
    if (count >= 6)
        // Long pulse - Yellow or Cyan
        return 3;

    // Nothing recognised
    return 0;
}

uint32_t last_update = 0; //timer for periodic Kilobot instruction

int direction_before=4; //initiate to something that is not 0-3

int random_walk=1; // random walk behavior on/off
int avoid_behavior = 1; //avoidance behavior on/off

uint32_t avoid_timer = 0; //avoidance timer

uint32_t random_duration = 0;
uint32_t random_timer = 0;

int behavior_complete=1;

void setup() {
    // put your setup code here, to be run only once
    last_update     = kilo_ticks;
    avoid_timer     = kilo_ticks;
}

void loop() {
    … //as for simulator

}

int main() {
    // initialize hardware
    kilo_init();
    // start program
    kilo_start(setup, loop);
    return 0;
}
```
